# Technically measured compositional physical work demands and prospective register-based sickness absence (PODESA): a study protocol

**DOI:** 10.1186/s12889-019-6581-z

**Published:** 2019-03-04

**Authors:** Sofie Dencker-Larsen, Charlotte Lund Rasmussen, Sannie Vester Thorsen, Els Clays, Thomas Lund, Merete Labriola, Ole Steen Mortensen, Marie Birk Jørgensen, Nidhi Gupta, Charlotte Diana Nørregaard Rasmussen, Andreas Holtermann

**Affiliations:** 1 National Research Centre for the Working Environment, Lersø Parkallé 105, Copenhagen, Denmark; 2 Section of Social Medicine, University of Copenhagen, Øster Farimagsgade 5, 1014, Copenhagen, Denmark; 3 Department of Public Health and Primary Care, Ghent University, 4K3 (ingang 42), Corneel Heymanslaan 10, B–9000 Ghent, Belgium; 4 Center for Social Medicine, Frederiksberg and Bispebjerg Hospital, Nordre Fasanvej 57, 2000 Frederiksberg, Denmark; 5 Department of Public Health, Aarhus University, Bartholins Allé 2, 8000 Aarhus, Denmark; 6 Department of Occupational and Social Medicine, Holbæk Hospital, Smedelundsgade 60, 4300 Holbæk, Denmark; 7 Department of Forensic Science, University of Copenhagen, 2100 Copenhagen, Denmark; 8 Department of Sports Science and Clinical Biomechanics, University of Southern Denmark, Campusvej 55, 5230 Odense, Denmark

**Keywords:** Accelerometers, Compositional data analysis (CoDA), Physical activity at work, Sick-leave, Time-use epidemiology

## Abstract

**Background:**

Various physical work demands are shown to be associated with sickness absence. However, these studies have: (a) predominantly used self-reported data on physical work demands that have been shown to be inaccurate compared with technical measurements, (b) principally focused on various physical work demands in ‘isolation’, i.e. ignoring their co-dependency – compositional nature –, and (c) mainly used register data on long-term sickness absence. The present article describes the protocol of a study with the objective of investigating the association between technically measured compositional data on physical work demands and prospective long- and short-term register-based data on sickness absence.

**Methods:**

‘The technically measured compositional Physical wOrk DEmands and prospective association with register-based Sickness Absence study (PODESA)’ comprises data from two Danish cohorts (NOMAD and DPhacto) primarily on blue-collar workers. In the PODESA cohort, data on 1108 workers were collected at baseline (between 2011 and 2014). The cohort data comprise, e.g., self-reported information on descriptives, lifestyle, workday, and health, as well as accelerometer-based measurements of physical work demands (physical activity, movements, and postures). These baseline measurements are linked with prospective register-based data on sickness absence for up to four years after baseline. The prospective association between physical work demands and sickness absence will be analysed using a Compositional Data Analysis approach.

**Discussion:**

PODESA provides a unique possibility of unravelling which combinations of physical work demands are associated with prospective sickness absence. PODESA employs technically measured information on physical work demands (taking into account the compositionality of physical work demand data) and prospective sickness absence data. The findings from PODESA can be used to develop strengthened preventive interventions for sickness absence. Results are expected in 2019–2021.

## Background

Sickness absence is costly to workers, employers, and society. In a 2008 report, the Danish Ministry of Employment presents that the yearly cost of sickness absence corresponds at least 37 billion DKK in unproductive wages and sickness absence subsidies alone in Denmark [1, 2]. Moreover, sickness absence is a considerable risk factor for workers permanently exiting the labour market [3–5].

Physical work demands – physical activity, movements, and postures at work – are amongst the dominant causes of long-term sickness absence [6, 7]. Physical work demands such as stationary standing [7, 8], sitting [9, 10], forward bending of the trunk [7, 8, 11], and arm elevation [7, 8, 11] have been shown to be associated with sickness absence [6–8, 12, 13].

However, there are three overall issues with the research literature on physical work demands and sickness absence: 1) physical work demands have mainly been measured using self-reports, 2) the analytical methods used in previous studies on physical work demands have predominantly ignored the compositional nature of the data, and 3) studies analysing sickness absence using register data, have generally omitted short-term sickness absence.

First, previous studies on physical work demands included self-reported information on physical work demands [14, 15], that have been presented to be less accurate than technical measurements [16–20] (e.g., for sitting time). Such technical measurements are in this case accelerometers attached to the body of study participants; accelerometers use accelerations of the body [21] to measure physical activity, movements, and postures. Therefore, future studies investigating the association between physical work demands and sickness absence are likely to strengthen the field of research when using accelerometer measurements.

Second, the vast majority of existing studies analysing physical work demands have investigated the effect of each physical work demand ‘in isolation’ of other physical work demands. For example, by, e.g., investigating the health effects of sitting time without taking into account the time spent on all remaining demands such as standing, or resting. Time-use on various physical work demands is constrained or fixed by nature – summing up to 100% – (or for example 8 h). Therefore, the proportion of time spent on physical work demands carries relative information, is co-dependent. Addressing this special property of data on physical work demands requires special statistical methodology – Compositional Data Analysis (CoDA) [22–25]. Only recently, a limited amount of studies have used Compositional Data Analysis approaches to address the compositional property of physical work demands [24, 26]. However, none of them have investigated the association between physical work demands and sickness absence. Therefore, future studies investigating the association between time spent on various physical work demands and prospective sickness absence using a Compositional Data Analysis approach are needed.

Third, the previous studies have often used sickness absence using self-reports that have less validity than sickness absence information from national registers in Nordic countries [27]. Studies using national register-data on sickness absence have predominantly used long-term sickness absence (see, e.g., [8, 12, 28–30]). Nevertheless, physical work demands has also been associated with short-term sickness absence [31], and, like sickness absence overall [32–35], is likely to be placing a considerable economic burden on workplaces and society. Studies investigating both long-term and short-term sickness absence register data are thus warranted.

### Aim

The purpose of the present article is to present the protocol for the ‘The technically measured compositional Physical wOrk DEmands and prospective association with register-based Sickness Absence study (PODESA)’. Specifically, to counter the above-presented challenges of previous studies, PODESA will be the first study to investigate the association between technically measured compositional data on physical work demands and prospective register-based data on short- and long-term sickness absence. PODESA will investigate the following hypothesis:

The composition of physical work demands is associated with prospective sickness absence.

## Design

### Data

PODESA will combine accelerometer-based data on physical work demands and other required data from the two Danish cohorts ‘New method for Objective Measurements of physical Activity in Daily living (NOMAD)’ [36] and ‘the Danish Physical Activity cohort with Objective measurements (DPhacto)’ [37]. These merged data will then be linked with prospective data on sickness absence using two types of extensive sickness absence registers.

### The PODESA cohort

The PODESA cohort consists of the merged NOMAD and DPhacto cohorts that were collected using almost identical data gathering procedures. Both cohorts included predominantly blue-collar workers from Denmark. Data collection on the NOMAD and DPhacto cohorts was conducted from 2011 to 2012 [16] and 2012 to 2014 [37], respectively. The NOMAD and DPhacto cohorts are described in detail elsewhere [37, 38]. In short, The NOMAD cohort included workers from seven workplaces within fields such as cleaning, construction, transport, and the health service sector [38], and the DPhacto cohort included workers from 15 workplaces within cleaning, manufacturing and transport industries [37, 39].

As shown in Table 1, we have combined three types of data from the NOMAD and DPhacto cohort into the PODESA cohort: 1) accelerometer data, 2) questionnaire data, and 3) health check data. The data from the PODESA cohort will be merged with detailed register data on sickness absence. At the time of submitting the manuscript, the authors had *not* yet conducted analyses of associations between exposure variables (PODESA cohort) and outcome variables (sickness absence data) (except, making the sample flowchart). This strategy was chosen to minimise bias of hindsight in the protocol.

**Table 1:** Data-merging strategy in PODESA

1) Constructing the PODESA cohort	
Data from the NOMAD and DPhacto cohorts have been combined into the PODESA cohort containing three types of data:	
Accelerometer data	Identical accelerometer hardware and software was used to measure physical work demands in both the NOMAD and in the DPhacto cohorts, making the two cohorts highly comparable; thus, the accelerometer data were added in a simple merge.
Questionnaire data	The majority of survey items from the questionnaires in the NOMAD and DPhacto cohorts are identical or comparable (77 items) enabling adding them in a simple merge. However, for the minority of survey items which were not identical, and merely similar, we assessed the comparability of the items and possible modification. Specifically, four items had accordance of question wording but dissimilar response scale size (e.g. nine versus ten categories), and five items that differed in wording (e.g. used non-identical time frames). A total of 14 items were non-comparable and therefore not merged. For similar, but not identical, survey items, the following procedure was used: firstly, based on findings from the literature within the field, we evaluated whether dissimilarities in wording or response scales could influence the answers, and secondly, using descriptive statistics we assessed the answer distribution in both cohorts. Similar items were merged if the literature indicated no difference in answers due to wording or response scale of the items *and* if the answer distribution on the items was similar in the two cohorts. The questionnaire data contain, e.g., background information to be used as covariates (such as age, sex, smoking status, alcohol intake). (An overview of the merging of the questionnaire data is available upon request).
Health check data	The health check data from the two studies derive from a health check and a physical testing session at baseline conducted by trained research professionals. Because identical health check procedures were followed in the NOMAD and DPhacto cohorts that are the basis of the PODESA cohort, we added the health check from each study in a simple merge. The health check data includes data on, e.g., height, weight, hip and waist circumference, percentage of body fat, blood pressure, maximal oxygen uptake, maximal hand grip strength, back extension endurance and back flexibility.
2) Combining the PODESA cohort with register data on sickness absence	
Data from the PODESA cohort will be linked with two types of register data on sickness absence	
We combine the PODESA cohort with register data from two registers:	
Register data on long-term sickness absence	In addition to the PODESA cohort data, we add register data on primarily long-tern sickness absence from the DREAM register dataset which includes weeks of subsidized sickness absence spells (typically granted after 30 days of sickness absence).
Register data on short-term sickness absence	We also add register data including short-term sickness absence from the ‘Danish Register of Work Absences’ which includes daily employer-reported sickness absence.

### Technical measurements of physical work demands

PODESA employs accelerometer-based data on physical work demands – physical activity, movements, and postures at work. Eligible participants of the cohort were asked to wear up to four triaxial ActiGraph accelerometers (GT3X+, Florida, U.S.A). The accelerometers were located at the dominant arm, upper back, hip, and right thigh. Participants wore the accelerometers for up to four consecutive workdays [36, 37]. Moreover, participants were asked to fill-in a short paper-based diary reporting time at work, time of going to bed and out of bed, non-wear time, and reference time (15 s of standing still to calibrate the accelerometer); and to remove the accelerometer if they experienced discomfort or itching [37]. Non-wear-time was determined by the following premises: (a) the software showed> 60 min of zero counts per minute, (b) the participant reported non-wear periods, and (c) visual inspection revealed artefacts or missing data [36].

The accelerometers were initialized and the data from the accelerometers were downloaded using the Actilife Software version 5.5 [40]. In brief, the accelerometer data are low-pass filtered with a 5 Hz 4th order Butterworth filter. Thereafter they were split-up into 2 s intervals with an overlap of 50% [36, 41].

Next, using a custom-made MATLAB program Acti4 [41, 42] (The National Research Centre for the Working Environment, Copenhagen, Denmark), the accelerometry data were analysed to obtain comprehensive information of physical work demands such as various physical activities (e.g., standing, walking, lying, cycling, stair climbing, and running) and postures (e.g., sitting, standing, forward bending, and arm elevation at various degrees). The Acti4 software has been shown to provide valid estimates of physical activities with high specificity and sensitivity> 99% under standardised and semi-standardized conditions [41]; in a free-living setting, Acti4 has been shown to have a specificity and sensitivity> 80% [43].

We will use the following specifications: We average daily time-use for all valid measured days on various physical work demands. Additionally, as physical work demands and physical activity behaviour at leisure are co-dependent [23], we expect to include information of time-use on various physical activity behaviours at leisure (i.e., sedentary behaviour, physical activity) and sleep domain (time in bed) summarized on all valid measured days. We consider a day to be valid if it comprises a valid work, leisure and sleep domain. A work and leisure period is considered valid if it comprises ≥4 h/day of accelerometer wear-time or ≥ 75% of the average wear-time across days (see, e.g., [24, 26, 44]). A time in bed period is considered valid if it comprises at least 4 h (see, e.g., [44]). To assess when participants were at work or at leisure or spent time-in-bed, we use information from the diaries.

### Measurements of sickness absence

PODESA will use data on sickness absence from two registers: 1) the DREAM register (comprising mainly long-term sickness absence) and 2) the ‘Danish register of work absences’ (covering a smaller portion of workers than the DREAM register, but includes also short-term sickness absence). Both are obtainable from Statistics Denmark [45–47].

First, the DREAM register is an acronym in Danish for ‘the Register-based Evaluation of Marginalization’ [3]). This Danish Ministry of Employment register is continuously updated and documented by The Danish Agency for Labour Market and Recruitment [48, 49]. It encompasses all episodes of sickness absence from work subsidized by the state. Typically these are granted after 30 days of sickness absence; thus excluding the often shorter periods of sickness absence which are not subsided. The DREAM register is widely used in studies measuring sickness absence (see, e.g., [8, 12, 28–30]).

Second, the ‘Danish Register of Work Absences’ (technical name used by Statistics Denmark: ‘FRAN’, ‘FRPE’) [45, 46, 50] includes sickness absence periods from work, including days on sickness absence [51]. This register contains data from all public employees and a considerable sample (*N* = 2600 companies) of privately owned companies, a representative sample of private companies with 10 to 249 employees, and all private companies with 250 or more employees. Private companies with 10 to 249 employees are sampled yearly (therefore, some workers at, e.g., midsize private companies are excluded from this register). Thus, this register includes shorter sickness absence spells for a limited amount of workers. Research studies using the ‘Danish Register of Work Absences’ are scarce (for such studies, please see [52, 53]).

From the above-mentioned registers, PODESA has access to prospective data on sickness absence since the worker baseline measurements (which vary between 2011 and 2014) with up until four years of follow-up sickness absence data (2011–2015).

### Study population

**Fig. 1:**
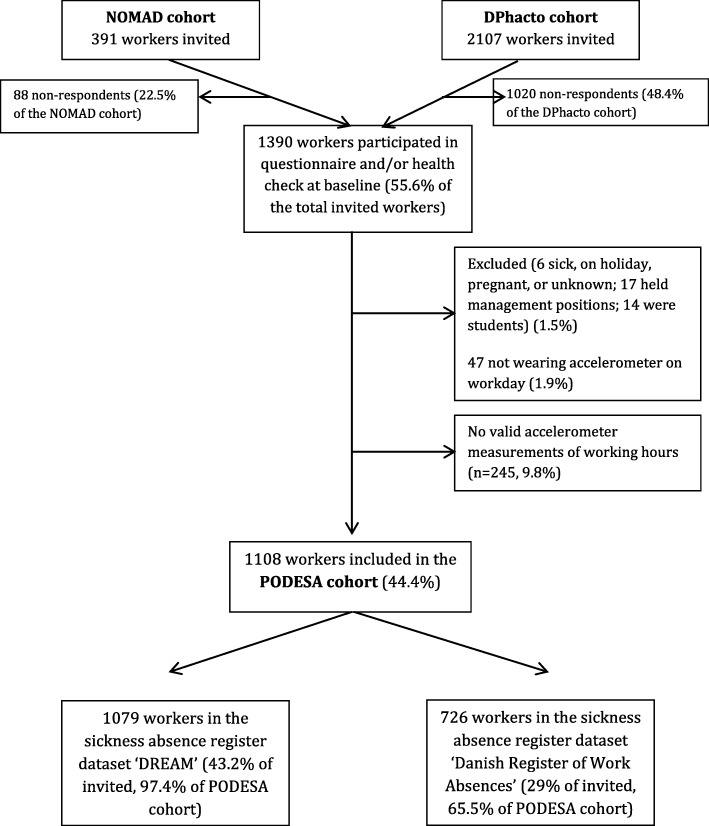
Flowchart of participants in PODESA with at least 1 year follow-up data

Figure 1 displays the number of participants in PODESA (*N* = 1108). A total of 391 and 2107 workers in the NOMAD cohort and the DPhacto cohort, respectively, were invited to participate. Of the 2498 invited participants, 1422 workers either handed in the questionnaire, underwent a health check at baseline, or both. Of these, a total of 1108 study participants fulfil the inclusion criteria and will be included in the PODESA cohort. Reasons for exclusion were: being sick, being on holiday, being pregnant, and ‘unknown’ (*N* = 6); holding a management position (*N* = 17), being a student (*N* = 14), and not wearing the accelerometers on a workday (*N* = 47).

Furthermore, the PODESA cohort will be linked with data on sickness absence for up to four years of follow-up. In the flowchart in Fig. 1, we *base the flowchart N* on workers with at least one year follow-up data from the sickness absence registers. Of the *N* = 1108 included workers in the PODESA cohort, there is sickness absence data on *N* = 1079 workers from the DREAM register, and *N* = 726 workers from ‘The Danish Register of Work Absences’.

## Statistical analyses

We expect to analyse the association between time-use in various physical work demands and prospective long-term sickness absence primarily using regression models such as time-to-event analyses based on a Compositional Data Analysis approach. This entails several steps: First, we will transform the 24-h compositional data on physical work demands and physical activity behaviour at leisure using an appropriate log-ratio method. Second, depending on the analytical definition of the outcome, we will adopt time-to-event methods (as the main analyses, we expect to use Cox time-to-event regression on sickness absence data from the DREAM sickness absence register). The models will be adjusted for variables such as age, sex, body mass index, smoking status (for similar covariates, please see, e.g., [8]). Third, to enable understanding how time spent in various postures and movements at work is associated with prospective sickness absence, we expect to use isotemporal substitution models [54] indicating association between reallocation of time-use in various physical work demands and the change in probability of prospective sickness absence.

Moreover, having access to detailed longitudinal register data from the two types of sickness absence registers enables us to code the outcome variable in several ways, such as binary, percentage of sickness absence, trajectories, and time-to-event, thus also enabling additional analyses further unravelling the relationship between physical work demands and sickness absence.

## Discussion

The PODESA study comprises high quality data, including technical measurements of physical work demands from accelerometer data, which provide a more precise depiction compared with, e.g., self-reports. Furthermore, the combined PODESA cohort data and sickness absence register data enable us to conduct analyses not only on the links between singular exposures and sickness absence, but also on the association between relative time-use on specific physical work demands and prospective sickness absence, e.g., using Compositional Data Analysis. Additionally, having access to prospective sickness absence data on both short-term and long-term sickness absence enables us to exploit the longitudinal nature of the data in analyses using, for example, trajectories or time-to-event analyses.

Conversely, there are also potential weaknesses to discuss. First, despite having high-quality data on physical work demands, the PODESA cohort has no objectively measured data on changes in physical work demands over time; if there is a non-random change, e.g., due to an organizational change or change in worker instructions at the workplace, it could affect the prevalence and timing of prospective sickness absence. Second, as the cohort data stem from a Danish context, generalizability of findings to other countries in terms of, e.g., inter-country variations in occupational policy is limited. Third, as the majority of participants in the study are blue-collar workers, the findings will primarily be generalizable to this group of workers.

The findings from PODESA can be used to develop improved preventive workplace interventions for sickness absence. For instance, if the results show which combinations of physical work demands may increase – and which may decrease – the risk of sickness absence, this information can be used to design better future preventive workplace interventions for sickness absence. Results from PODESA are expected in 2019–2021.

## Abbreviations

DPhacto: The Danish Physical Activity cohort with Objective measurements
DREAM: A Danish acronym for ‘the Register-based Evaluation of Marginalization
NOMAD: New method for Objective Measurements of physical Activity in Daily living
PODESA: The technically measured compositional Physical wOrk DEmands and prospective association with register-based Sickness Absence study
